# Blood Metal Levels and Amyotrophic Lateral Sclerosis Risk: A Prospective Cohort

**DOI:** 10.1002/ana.25932

**Published:** 2020-11-06

**Authors:** Susan Peters, Karin Broberg, Valentina Gallo, Michael Levi, Maria Kippler, Paolo Vineis, Jan Veldink, Leonard van den Berg, Lefkos Middleton, Ruth C. Travis, Manuela M. Bergmann, Domenico Palli, Sara Grioni, Rosario Tumino, Alexis Elbaz, Tim Vlaar, Francesca Mancini, Tilman Kühn, Verena Katzke, Antonio Agudo, Fernando Goñi, Jesús‐Humberto Gómez, Miguel Rodríguez‐Barranco, Susana Merino, Aurelio Barricarte, Antonia Trichopoulou, Mazda Jenab, Elisabete Weiderpass, Roel Vermeulen

**Affiliations:** ^1^ Institute for Risk Assessment Sciences Utrecht University Utrecht the Netherlands; ^2^ Department of Neurology University Medical Center Utrecht Utrecht the Netherlands; ^3^ Institute of Environmental Medicine Karolinska Institute Solna Sweden; ^4^ Centre for Primary Care and Public Health Queen Mary University of London London UK; ^5^ School of Public Health Imperial College London London UK; ^6^ Cancer Epidemiology Unit Nuffield Department of Population Health, University of Oxford Oxford UK; ^7^ German Institute of Human Nutrition Nuthetal Germany; ^8^ Cancer Risk Factors and Life‐Style Epidemiology Unit Institute for Cancer Research, Prevention and Clinical Network Florence Italy; ^9^ Epidemiology and Prevention Unit IRCCS National Cancer Institute Foundation Milan Italy; ^10^ Cancer Registry and Histopathology Department Provincial Health Company Ragusa Italy; ^11^ Public Health France Saint‐Maurice France; ^12^ Paris‐Sud University UVSQ, CESP, INSERM, Paris‐Saclay University Villejuif France; ^13^ Faculty of Medicine, CESP, Paris‐Sud University, UVSQ, INSERM Paris‐Saclay University Villejuif France; ^14^ Gustave Roussy Institute Villejuif France; ^15^ German Cancer Research Center Heidelberg Germany; ^16^ Unit of Nutrition and Cancer, Cancer Epidemiology Research Program, Catalan Institute of Oncology, Group of Research on Nutrition and Cancer Bellvitge Biomedical Research Institute, L'Hospitalet de Llobregat Barcelona Spain; ^17^ Networked Biomedical Research Center of Epidemiology and Public Health Madrid Spain; ^18^ Biodonostia Health Research Institute; Public Health Laboratory in Gipuzkoa Basque Government San Sebastian Spain; ^19^ Department of Epidemiology Regional Health Council, IMIB‐Arrixaca Murcia Spain; ^20^ Andalusian School of Public Health Granada Spain; ^21^ Grenada Institute of Biosanitary Research Granada Spain; ^22^ Public Health Directorate Asturias Spain; ^23^ Navarra Public Health Institute Pamplona Spain; ^24^ Navarra Institute for Health Research Pamplona Spain; ^25^ Hellenic Health Foundation Athens Greece; ^26^ International Agency for Research on Cancer Lyon France; ^27^ Julius Center for Health Sciences and Primary Care University Medical Center Utrecht Utrecht the Netherlands

## Abstract

**Objective:**

Metals have been suggested as a risk factor for amyotrophic lateral sclerosis (ALS), but only retrospective studies are available to date. We compared metal levels in prospectively collected blood samples from ALS patients and controls, to explore whether metals are associated with ALS mortality.

**Methods:**

A nested ALS case–control study was conducted within the prospective EPIC (European Prospective Investigation into Cancer and Nutrition) cohort. Cases were identified through death certificates. We analyzed metal levels in erythrocyte samples obtained at recruitment, as a biomarker for metal exposure from any source. Arsenic, cadmium, copper, lead, manganese, mercury, selenium, and zinc concentrations were measured by inductively coupled plasma–mass spectrometry. To estimate ALS risk, we applied conditional logistic regression models.

**Results:**

The study population comprised 107 cases (65% female) and 319 controls matched for age, sex, and study center. Median time between blood collection and ALS death was 8 years (range = 1–15). Comparing the highest with the lowest tertile, cadmium (odds ratio [OR] = 2.04, 95% confidence interval [CI] = 1.08–3.87) and lead (OR = 1.89, 95% CI = 0.97–3.67) concentrations suggest associations with increased ALS risk. Zinc was associated with a decreased risk (OR = 0.50, 95% CI = 0.27–0.94). Associations for cadmium and lead remained when limiting analyses to noncurrent smokers.

**Interpretation:**

This is the first study to compare metal levels before disease onset, minimizing reverse causation. The observed associations suggest that cadmium, lead, and zinc may play a role in ALS etiology. Cadmium and lead possibly act as intermediates on the pathway from smoking to ALS. ANN NEUROL 20209999:n/a–n/a

Amyotrophic lateral sclerosis (ALS) is a relentlessly progressive and fatal neurodegenerative disease involving the central and peripheral nervous system of as yet unknown etiology. The disease is characterized by loss of motor neurons, resulting in progressive muscle weakness, swallowing difficulties, and finally death due to respiratory failure. Most patients die within 3 to 4 years after the first symptoms, although survival is variable.^1^ ALS is the most common adult onset motor neuron disease, with an estimated incidence of 2.6 to 3.0 per 100,000 person‐years.^1^

Exposure to metals has been suggested as a possible risk factor for ALS for many years.^2^, ^3^ Exposures may occur in a wide range of occupational settings, for example, welding, plumbing, manufacturing, metal smelting, and refining, but also occur in the general population through contaminated food or drinking water, air pollution, cigarette smoking, medication, or dietary supplements.^4^, ^5^

Previous studies on the association between metal exposure and ALS risk were limited by cross‐sectional or case–control designs, where metal concentrations were measured only after disease onset, for example.^6^, ^7^, ^8^ As such, these studies did not allow inference on temporality and were unable to rule out reverse causality. Observed differences in blood metal levels in ALS patients could be the consequence of disease progression, rather than representing pre–disease exposure levels. For example, reduced physical activity resulting from widespread muscle weakness (a key clinical feature of ALS) may give rise to an increase in bone turnover, leading to a raised release of lead blood levels. Metals have also been found in the locus coeruleus and motor neurons of ALS patients, but the significance of this finding in disease etiology remains unclear.^9^

To determine whether an increased burden of metals contributes to the etiopathogenic mechanisms leading to ALS, and does not result from the disease, prospective data are needed. A prospective design, however, is challenging for relatively rare diseases such as ALS. The European Prospective Investigation into Cancer and Nutrition (EPIC) cohort, with >500,000 subjects and a follow‐up time of up to 15 years, offered a unique opportunity to compare prediagnostic blood metal levels of ALS cases and controls. Here, for the first time, results from an ALS case–control study nested in a prospective cohort are presented. We hypothesize that ALS patients have increased blood metal levels before disease onset, including the neurotoxicants lead, manganese, and mercury, and possibly also arsenic, cadmium, copper, selenium, and zinc.

## Subjects and Methods

### *Study Population*

A nested case–control study was conducted within the EPIC cohort.^10^, ^11^ This cohort is a large prospective study initiated in 1992 that was designed to investigate the relationship between diet, lifestyle, and environmental factors and the incidence of cancer and other chronic diseases. Recruitment took place between 1993 and 1999. In total, more than half a million (520,000) people, with the vast majority aged between 35 and 70 years, were recruited in 10 European countries: Denmark, France, Germany, Greece, Italy, the Netherlands, Norway, Spain, Sweden, and the United Kingdom. Data on diet and lifestyle were obtained through structured questionnaires at baseline. At the same time, anthropometric measurements and blood samples were taken.

The EPIC study was approved by the ethical committee of the International Agency for Research on Cancer (IARC) and by the ethical review boards of each participating center. All participants signed an informed consent.

ALS cases were defined as those subjects for whom “motor neuron disease” (G12.2 according to the 10th revision of the International Classification of Diseases) was reported as an immediate, antecedent, or underlying cause of death. In this and other ALS studies, mortality has been shown to be a good proxy for disease incidence, given the almost invariable 100% fatality rate and the relatively short duration of the disease.^12^ A total of 168 people who died from ALS had been identified within the EPIC cohort after a median follow‐up time of 8 years (range = 1–15). Blood samples from the Nordic countries were not available in the central biobank, resulting in 107 cases included for analyses. Three controls per case were selected by incidence density sampling matched by age at recruitment, sex, and study center (to account for center‐specific differences in questionnaire design, blood collection procedures, etc).

### *Biological Samples*

A 30ml sample of blood was obtained from each participant at recruitment into the study. Plasma, serum, erythrocytes, and buffy coat were separated by centrifugation, aliquoted into 0.5ml plastic straws, and stored in liquid nitrogen at −196°C until they were chosen for analyses. Metals are to a large extent bound to erythrocytes in blood. Given the relatively long lifetime of erythrocytes (around 3 months), erythrocyte concentrations of these metals have been shown to be a relevant biomarker of ongoing exposure.^4^

The erythrocyte samples were prepared for inductively coupled plasma–mass spectrometry (ICP‐MS) analysis by a direct alkali dilution method.^13^ Briefly, samples were diluted 1:25–50 with an alkali solution consisting of 2% butanol (Honeywell Research Chemicals, Seelze, Germany), 0.05% ethylenediaminetetraacetic acid (Sigma‐Aldrich, St Louis, MO), 0.05% Triton X‐100 (Sigma‐Aldrich), 1% NH_4_OH (Romil, Cambridge, UK), and 20μg/g internal standards. Before analysis, the diluted samples were centrifuged at 2,000rpm for 5 minutes.

The Agilent 7700x ICP‐MS (Agilent Technologies, Tokyo, Japan) equipped with an octopole reaction system collision/reaction cell technology was used for measuring concentrations of 8 elements (isotope): arsenic (^75^As), cadmium (^111^Cd), copper (^63^Cu), lead (^208^Pb), manganese (^55^Mn), mercury (^202^Hg), selenium (^78^Se), and zinc (^66^Zn). The elements germanium (^72^Ge), rhodium (^103^Rh), lutethium (^175^Lu), and iridium (^193^Ir) were included as internal standards. The majority of the elements were measured in helium mode (^55^Mn, ^63^Cu, ^66^Zn, ^75^As, ^111^Cd; gas flow = 5ml/min), whereas ^78^Se was measured in hydrogen mode (gas flow = 5–6ml/min) and ^202^Hg and ^208^Pb in standard mode (no gas). One part per million of a gold standard (Sigma‐Aldrich) was added to all solutions to stabilize mercury.

The limit of detection (LOD) for each element was determined as 3 × standard deviation (SD) of analyzed blanks (alkali solution) and as signal/noise = 3. The limit of quantification (LOQ) was determined as 10 × SD of analyzed blanks. As quality control, 2 commercially available whole blood reference materials, Seronorm level‐1 WB, lot 1702821 and Seronorm level‐2 WB, lot 1406264 (SERO, Billingstad, Norway), were analyzed (Supplementary Table S1). Blanks and reference materials were treated together with the collected erythrocyte samples and analyzed in the beginning, in the middle, and at the end of each analysis.

### *Statistical Analyses*

Values of metal concentrations below the LOD were replaced by LOD/√2 (n = 1 [0.2%] for cadmium and n = 9 [2.1%] for mercury). No values between LOD and LOQ were obtained. Metal levels were log‐transformed to normalize their distribution.

Tertiles of metal concentrations were calculated based on the distribution among controls. Conditional logistic regression models for the matched case–control sets were applied to estimate the odds ratio (OR) and 95% confidence interval (CI) of ALS in relation to each of the blood metal levels before disease onset. Probability values for linear and flexible spline trends were based on the continuous levels. We examined cigarette smoking, body mass index, physical activity, and alcohol consumption as possible confounding factors, but these variables did not modify the risk estimates (*p* > 0.1) and were therefore not included in the models. We also examined educational level as a possible confounder, because lower education has been suggested to be associated with higher risk of ALS, although this association was largely explained by smoking.^14^ In our data, educational level was highly unbalanced across study centers, and we observed no consistent association. As such, we decided not to adjust for education in our models.

We performed several sensitivity analyses to test the robustness of our findings, as follows: (1) exclude cases who died within 3 years (median survival time from symptom onset) of recruitment to further minimize possible reverse causation; (2) exclude current smokers at baseline, because cigarette smoking is a recognized source of metals and a risk factor for ALS^12^, ^15^; and (3) exclude ever‐smokers. For the analyses on noncurrent and never‐smokers, unconditional logistic regression models were applied to preserve statistical power, adjusted for the matching variables sex, age at recruitment, and study center.

Exposure to metals may result, among other sources, from cigarette smoking or dietary intake (eg, methylmercury and selenium from fish and selenium and cadmium from cereals). We therefore looked at the correlations between metal levels to identify patterns in our data (with smoking status or intakes of fish/shellfish and cereal).

All statistical analyses were carried out in SAS v9.4 (SAS Institute, Cary, NC).

## Results

The study population comprised 107 cases (65% female) and 319 controls (Table 1). The median age at recruitment was 60 years, and the median time between blood collection at recruitment and ALS death was 8 years (ranging from 1 to 15 years). Among the controls, 13% were smokers at baseline, compared with 16% among cases.

**TABLE 1:** Baseline Characteristics of the ALS Cases and Controls within the EPIC Cohort

Characteristic	Controls, n = 319	ALS Cases, n = 107
Age at recruitment, yr, median (range)^a^	60.5 (34.6–76.1)	60.4 (35.0–75.2)
Years between recruitment and ALS death, median (range)	—	8.1 (1.0–15)
Sex, n (%)^a^		
Male	110 (34%)	37 (35%)
Female	209 (66%)	70 (65%)
Country, n (%)^a^		
France	33 (10%)	11 (10%)
Italy	36 (11%)	12 (11%)
Spain	50 (16%)	17 (16%)
United Kingdom	93 (29%)	31 (29%)
The Netherlands	57 (18%)	19 (18%)
Greece	9 (2.8%)	3 (2.8%)
Germany	41 (13%)	14 (13%)
Educational level, n (%)		
None/primary	106 (33%)	50 (47%)
Technical	72 (23%)	14 (13%)
Secondary	47 (15%)	17 (16%)
University	61 (19%)	20 (19%)
Unknown	33 (10%)	6 (5.6%)
Smoking status at recruitment, n (%)		
Never‐smoker	164 (51%)	53 (50%)
Former smoker	104 (33%)	35 (33%)
Current smoker	43 (13%)	17 (16%)
Unknown	8 (2.5%)	2 (1.9%)

^a^ Matching variables.

ALS = amyotrophic lateral sclerosis.

Table 2 shows the erythrocyte metal concentrations (ng/g) for both cases and controls. Correlations between metal concentrations as well as with possible determinants of exposure are presented in Table 3. Exploration of possible sources of metals indicated correlations between particularly cadmium and to a lesser extent lead levels with cigarettes per day, as well as arsenic, mercury, and selenium levels with fish/shellfish consumption. No correlation was observed between metal concentrations and cereal consumption. Among cases, no association between the metal concentrations and time to ALS death was observed (data not shown).

**TABLE 2:** Descriptive Statistics of Erythrocyte Metal Levels (ng/g) for ALS Cases and Controls

	Controls, n = 319					ALS Cases, n = 107				
Metal	AM	GM	GSD	Min–Max	<LOD, n	AM	GM	GSD	Min–Max	<LOD, n
Arsenic	4.74	2.83	2.83	0.27–83.5	0	5.22	2.68	3.20	0.25–43.3	0
Cadmium	0.90	0.70	2.11	0.002–4.94	1	0.98	0.78	1.89	0.22–8.43	0
Copper	592	587	1.14	402–1,042	0	585	580	1.15	381–828	0
Lead	84.4	72.7	1.68	17.4–688	0	87.4	78.1	1.63	22.1–240	0
Manganese	14.5	13.9	1.35	5.33–31.1	0	14.5	13.7	1.39	7.45–34.8	0
Mercury	5.44	2.75	4.29	0.004–55.8	8	5.03	2.82	3.57	0.004–30.1	1
Selenium	123	117.7	1.31	56.4–670	0	118	115	1.25	62.0–230	0
Zinc	9,090	8,941	1.20	5,024–18,411	0	8,817	8,697	1.18	5,136–12,577	0

ALS = amyotrophic lateral sclerosis; AM = arithmetic mean; GM = geometric mean; GSD = geometric standard deviation; LOD = limit of detection.

**TABLE 3:** Pearson Correlation Coefficients between Blood Metal Concentrations and Possible Sources of Metal Exposure

Correlations between Blood Metal Concentrations								Correlation with Possible Sources		
	Cd	Cu	Pb	Mn	Hg	Se	Zn	Cigarettes per Day	Cereal Consumption	Fish/Shellfish Consumption
As	−0.04	0.05	0.08	0.07	0.62	0.40	0.14	−0.05	−0.01	0.36
Cd		−0.02	0.15	0.07	−0.03	−0.07	0.01	0.35	−0.01	−0.05
Cu			−0.07	0.15	0.01	0.11	0.31	0.02	−0.09	−0.05
Pb				−0.05	0.14	0.05	0.08	0.11	0.05	0.02
Mn					0.08	0.13	0.08	−0.12	−0.02	0.03
Hg						0.42	0.10	−0.01	−0.04	0.38
Se							0.24	0.06	0.08	0.27
Zn								−0.02	0.06	−0.07

As = arsenic; Cd = cadmium; Cu = copper; Hg = mercury; Mn = manganese; Pb = lead; Se = selenium; Zn = zinc.

Logistic regression models suggested that cadmium and lead levels were associated with an increased risk of ALS (Table 4). The ORs for tertiles of cadmium levels were 2.16 (95% CI = 1.18–3.97) and 2.04 (95% CI = 1.08–3.87) for the second and third tertile compared to the first, respectively. The corresponding ORs for lead were 1.83 (95% CI = 0.99–3.35) and 1.89 (95% CI = 0.97–3.67), respectively. No strong trends on the continuous scale were observed. In contrast, zinc levels were associated with decreased risk for ALS (highest vs lowest tertile: OR = 0.50, 95% CI = 0.27–0.94).

**TABLE 4:** Conditional Logistic Regression Models on the Association between Blood Metal Concentrations and the Risk of Amyotrophic Lateral Sclerosis

Metal	Exposure Category, ng/g	Controls, n	Cases, n	Total Group, OR (95% CI)	Excluding Cases Who Died within 3 Years, n = 13, OR (95% CI)
Arsenic	≤1.67	105	39	1.00 [Ref]	1.00 [Ref]
	>1.67 ≤ 4.37	105	29	0.71 (0.38–1.32)	0.68 (0.35–1.33)
	>4.37	109	39	0.90 (0.47–1.72)	0.78 (0.39–1.56)
	*p* for trend, linear			0.517	0.296
	*p* for trend, spline			0.198	0.110
Cadmium	≤0.51	105	22	1.00 [Ref]	1.00 [Ref]
	>0.51 ≤ 0.91	105	44	2.16 (1.18–3.97)	2.35 (1.21–4.56)
	>0.91	109	41	2.04 (1.08–3.87)	1.94 (0.96–3.92)
	*p* for trend, linear			0.122	0.261
	*p* for trend, spline			0.498	0.739
Copper	≤553	106	38	1.00 [Ref]	1.00 [Ref]
	>553 ≤ 610	104	28	0.77 (0.44–1.37)	0.70 (0.38–1.28)
	>610	109	41	1.07 (0.59–1.96)	1.07 (0.57–2.00)
	*p* for trend, linear			0.360	0.310
	*p* for trend, spline			0.738	0.693
Lead	≤56.8	105	25	1.00 [Ref]	1.00 [Ref]
	>56.8 ≤ 89.0	105	41	1.83 (0.99–3.35)	1.92 (0.99–3.73)
	>89.0	109	41	1.89 (0.97–3.67)	1.82 (0.88–3.73)
	*p* for trend, linear			0.153	0.176
	*p* for trend, spline			0.297	0.380
Manganese	≤12.5	106	42	1.00 [Ref]	1.00 [Ref]
	>12.5 ≤ 15.8	104	35	0.87 (0.52–1.46)	0.93 (0.54–1.63)
	>15.8	109	30	0.69 (0.40–1.19)	0.59 (0.33–1.07)
	*p* for trend, linear			0.780	0.489
	*p* for trend, spline			0.092	0.070
Mercury	≤1.84	106	36	1.00 [Ref]	1.00 [Ref]
	>1.84 ≤ 4.82	104	33	0.94 (0.54–1.64)	0.86 (0.47–1.57)
	>4.82	109	38	1.06 (0.53–2.11)	1.10 (0.53–2.28)
	*p* for trend, linear			0.879	0.861
	*p* for trend, spline			0.344	0.510
Selenium	≤104	109	32	1.00 [Ref]	1.00 [Ref]
	>104 ≤ 123	102	38	1.31 (0.74–2.31)	1.39 (0.76–2.55)
	>123	108	37	1.21 (0.65–2.25)	1.31 (0.67–2.56)
	*p* for trend, linear			0.374	0.487
	*p* for trend, spline			0.850	0.960
Zinc	≤8,425	106	48	1.00 [Ref]	1.00 [Ref]
	>8,425 ≤ 9,566	105	29	0.54 (0.30–0.97)	0.50 (0.27–0.94)
	>9,566	108	30	0.50 (0.27–0.94)	0.50 (0.25–0.98)
	*p* for trend, linear			0.100	0.206
	*p* for trend, spline			0.760	0.792

CI = confidence interval; OR = odds ratio; Ref = reference.

Sensitivity analyses excluding patients who died within 3 years of recruitment (n = 13) revealed virtually the same results (see Table 4). An interaction of metal levels with smoking status and the risk for ALS was observed for cadmium (*p* = 0.009) but not for lead (*p* = 0.626). The observed associations for both cadmium and lead remained when excluding current smokers at baseline, showing a clear linear trend for cadmium (*p* = 0.023; Table 5). These associations weakened when further limiting the models to never‐smokers, but the numbers became small. No interactions between zinc and cadmium or zinc and lead were observed.

**TABLE 5:** Unconditional Logistic Regression Models on the Association between Blood Metal Concentrations and the Risk of Amyotrophic Lateral Sclerosis, Limited to Noncurrent Smokers and Never‐Smokers^a^

	Exposure Category, ng/g	Noncurrent Smokers			Never‐Smokers		
		Controls, n	Cases, n	OR^b^ (95% CI)	Controls, n	Cases, n	OR^b^ (95% CI)
Cadmium	≤0.51	102	19	1.00 [Ref]	59	14	1.00 [Ref]
	>0.51 ≤ 0.91	97	38	2.16 (1.14–4.09)	62	26	1.82 (0.83–4.03)
	>0.91	69	31	2.62 (1.32–5.22)	43	13	1.38 (0.54–3.54)
	*p* for trend, linear			0.023			0.503
Lead	≤56.8	91	22	1.00 [Ref]	56	14	1.00 [Ref]
	>56.8 ≤ 89.0	92	38	1.86 (0.99–3.48)	61	24	1.83 (0.79–4.24)
	>89.0	85	28	1.62 (0.81–3.26)	47	15	1.55 (0.60–4.01)
	*p* for trend, linear			0.178			0.637
Zinc	≤8,425	88	37	1.00 [Ref]	57	21	1.00 [Ref]
	>8,425 ≤ 9,566	93	25	0.60 (0.32–1.12)	49	15	0.82 (0.36–1.87)
	>9,566	95	28	0.68 (0.36–1.29)	58	17	0.84 (0.36–1.94)
	*p* for trend, linear			0.254			0.460

^a^ Smoking status was missing for 2 cases and 8 controls, who were excluded from these analyses.

^b^ Odds ratio adjusted for matching variables sex, age at recruitment and center.

CI = confidence interval; OR = odds ratio; Ref = reference.

## Discussion

Our study is the first prospective study investigating blood metal levels and the risk of ALS to date. The metal concentrations in blood from ALS patients, before disease onset, and controls indicated that lead and cadmium may be associated with an increased risk of ALS, with evidence of an inverse association for zinc.

Previously observed positive associations between lead level and risk of ALS were based on the comparison of (post–disease onset) blood levels,^6^, ^7^, ^16^ as well as on assessments of occupational exposure to lead, using registry data.^17^ Our data support these observations. The relatively small magnitude of increase in risk observed for lead in our study may reflect the multifactorial nature of ALS, in which lead exposure is one of the potential steps in the multistage etiologic process leading to the disease.^18^ Among relevant etiologic factors, genetic variants have been discussed in the literature, such as the highly penetrant *C9orf72* repeat expansion. The *C9orf72* repeat expansion is present in about 8% of the sporadic ALS cases in European populations,^19^ but unfortunately, we had no information on genetic variants.

Further associations between other metals and the risk of ALS have also been reported,^3^ but much less studied. Our new findings, particularly for cadmium and zinc, warrant further research.

Cigarette smoking is one of the few established risk factors for ALS, which also appeared to be a risk factor in our study population.^12^ The mechanisms or the causing components of cigarette smoke for the development of ALS, however, are still unclear. Cigarette smoking is a recognized source of metals; the tobacco plant takes up cadmium,^20^ which is inhaled by the smokers, and smokers were shown to have higher cadmium blood levels than nonsmokers.^21^ Smoking is also associated with higher blood lead levels and with lower selenium and zinc levels.^22^ This pattern was also reflected in our data. We nevertheless observed the same associations between blood metal levels and ALS risk among participants who gave up smoking or never have smoked cigarettes, suggesting a possible role of cadmium and lead in ALS etiology, independent of cigarette smoking. Future studies should explore mechanisms of actions by conducting formal mediation analysis.

Our study is the first ever to investigate metal levels in blood samples from ALS patients collected prior to disease and to compare these samples to those from matched controls from the same source population. The prospective design of the EPIC study allowed an insight on temporality, arguing against reverse causality, which may be suspected based on results of classical case–control studies. Similar to the blood samples, the data on lifestyle factors such as smoking have also been collected before the onset of disease, ruling out the distortion of risk estimates by recall bias. The lack of association between metal levels and time to ALS death confirms this assumption.

Survival of ALS is highly variable, ranging from a few months to >10 years.^1^ In our study population, we had no clinical data to confirm the time of onset. As such, we cannot be sure that all included samples were truly presymptomatic. Sensitivity analyses limited to those who died >3 years after recruitment produced similar results, but also a 3‐year cutoff remains arbitrary. Some excluded participants may have been without symptoms at time of blood collection, and among those who remained in the analyses there may have been long survivors for whom the disease process had already started before recruitment. Furthermore, although EPIC is a large cohort, our overall number of ALS cases was not high due to the rarity of the disease. Our study would have benefitted from a larger number of cases, which could be achieved by extended follow‐ups of mortality in the future. Larger numbers would also have allowed for further restricting analyses by time until death.

The life span of erythrocytes is typically 120 days in healthy adults.^23^ The observed metal concentrations therefore reflect only exposure close to the time point when the blood samples were collected. The observed concentrations, however, will be correlated to the overall retention of the metals in the body, unless people drastically changed their exposure conditions. The latter is unlikely, because we analyzed blood samples from subjects before disease onset. As such, our data will have well reflected the relative ranking of exposure levels of both cases and controls. Possibly, we have underestimated occupational exposures to metals, which may have been higher in the more distant past. Given the typically low prevalence of workers with high metal exposure in the general population,^24^ however, our results are unlikely to be affected by missing possible occupational exposures.

The coefficient of variation for cadmium was relatively high for the low‐level reference sample with 34% (see Supplementary Table S1), whereas the coefficients for the other elements were all below 15%. Cadmium showed somewhat unstable signals close to LOD. We therefore categorized the metal concentrations into tertiles, to avoid giving samples with low concentrations and less confident measurements too much weight.

In conclusion, the metal concentrations in blood from ALS patients (obtained before disease onset) and controls indicate that cadmium and lead may be associated with an increased risk of ALS and zinc with a decreased risk. This is the first study to evaluate predisease metal levels in blood, thus minimizing reverse causation. Although our results are inconclusive, we saw a positive association for lead, for which there was the strongest a priori evidence. Our observations suggest that these metals may play a role in ALS etiology and warrant further studies in other populations, although few cohorts have stored presymptomatic erythrocytes.

## Author Contributions

S.P., V.G., P.V., L.M., R.C.T., M.M.B., D.P., S.G., R.T., A.E., T.V., F.M., T.K., V.K., A.A., F.G., J.‐H.G., M.R.‐B., S.M., A.B., A.T., M.J., E.W., and R.V. contributed to the conception and design of the study; all authors contributed to the acquisition and analysis of data; S.P. and R.V. contributed to drafting the text.

## Potential Conflicts of Interest

Nothing to report.

## Supporting information


**SUPPLEMENTARY TABLE S1**. Accuracy^a^ and Precision^b^ of Inductively Coupled Plasma–Mass Spectrometry Method
