# Supplementary material for: Blood Metal Levels and Amyotrophic Lateral Sclerosis Risk: A Prospective Cohort
Source: Ann Neurol. 2020 Nov 6;89(1):125–33. doi: 10.1002/ana.25932 (PMC7756568; doi:10.1002/ana.25932)
Supplement: Supplementary file 1 — SUPPLEMENTARY TABLE S1. Accuracya and Precisionb of Inductively Coupled Plasma–Mass Spectrometry Method [file ANA-89-125-s001.docx]

Supplemental Table 1. Accuracy^a^ and precision^b^ of ICP-MS method

| Sample |  | As | Cd | Cu | Pb | Mn | Hg | Se | Zn |
| --- | --- | --- | --- | --- | --- | --- | --- | --- | --- |
| **Seronorm WB-1702821 L-1** |  |  |  |  |  |  |  |  |  |
| Measured (µg/kg) | Mean (n=33) | 2.0 | 0.32^d^ | 615 | 10.6 | 19.3 | 1.46 | 58 | 4168 |
|  | SD | 0.2 | 0.11 | 43 | 0.5 | 1.2 | 0.17 | 9 | 370 |
| Reference (µg/kg)^c^ |  | 2.0 | 0.27 | 607 | 9.5 | 18.7 | 1.49 | 65 | 4360 |
| SD^c^ |  | 0.4 | 0.06 | 57 | 1.9 | 1.5 | 0.30 | 14 | 758 |
| *Recovery* |  | *100%* | *119%* | *101%* | *112%* | *103%* | *98%* | *89%* | *96%* |
| *CV* |  | *10.7%* | *34.3%* | *7.0%* | *5.1%* | *6.1%* | *11.4%* | *14.8%* | *8.9%* |
| **Seronorm WB-1406264 L-2**  Measured (µg/kg) | Mean (n=34) | 12.3 | 4.99 | 1201 | 310 | 30.7 | 16.7 | 157 | 6865 |
|  | SD | 1.2 | 0.18 | 64 | 11 | 1.2 | 2.1 | 11 | 444 |
| Reference (µg/kg)^c^ |  | 13.4 | 4.75 | 1270 | 319 | 29.8 | 16.1 | 153 | 6730 |
| SD^c^ |  | 2.7 | 0.96 | 256 | 64 | 6.0 | 3.2 | 30 | 1327 |
| *Recovery* |  | *92%* | *105%* | *95%* | *97%* | *103%* | *103%* | *103%* | *102%* |
| *CV* |  | *9.4%* | *3.5%* | *5.3%* | *3.7%* | *3.9%* | *12.7%* | *7.0%* | *6.5%* |

^a^ Expressed as recovery%; ^b^ Expressed as CV%; ^c^ Certified values and standard deviation (SD) were converted from µg/L to µg/kg by division with the average density of blood 1.055 kg/L; ^d^ One day was excluded due to Cd contamination, n=30.

As = arsenic; Cd = cadmium; Cu = copper; Pb = lead; Mn = manganese; Hg = mercury; Se = selenium; Zn = zinc
